# Comparison of multiple machine learning models for predicting prognosis of pancreatic ductal adenocarcinoma based on contrast-enhanced CT radiomics and clinical features

**DOI:** 10.3389/fonc.2024.1419297

**Published:** 2024-11-13

**Authors:** Yue Huang, Han Zhang, Qingzhu Ding, Dehua Chen, Xiang Zhang, Shangeng Weng, Guozhong Liu

**Affiliations:** ^1^ Department of Hepatopancreatobiliary Surgery, The First Affiliated Hospital, Fujian Medical University, Fuzhou, Fujian, China; ^2^ Fujian Abdominal Surgery Research Institute, The First Affiliated Hospital of Fujian Medical University, Fuzhou, Fujian, China; ^3^ National Regional Medical Center, Binhai Campus of the First Affiliated Hospital, Fujian Medical University, Fuzhou, Fujian, China; ^4^ Department of Radiology, The First Affiliated Hospital of Fujian Medical University, Fuzhou, Fujian, China; ^5^ Fujian Provincial Key Laboratory of Precision Medicine for Cancer, The First Affiliated Hospital of Fujian Medical University, Fuzhou, Fujian, China; ^6^ Clinical Research Center for Hepatobiliary Pancreatic and Gastrointestinal Malignant Tumors Precise Treatment of Fujian, The First Affiliated Hospital of Fujian Medical University, Fuzhou, Fujian, China

**Keywords:** pancreatic ductal adenocarcinoma, machine learning, inflammatory marker, radiomics, prognosis

## Abstract

**Objective:**

The aim of this study was to evaluate the prognostic potential of combining clinical features and radiomics with multiple machine learning (ML) algorithms in pancreatic ductal adenocarcinoma (PDAC).

**Methods:**

A total of 116 patients with PDAC who met the eligibility criteria were randomly assigned to a training or validation cohort. Seven ML algorithms, including Supervised Principal Components, stepwise Cox, Random Survival Forest, CoxBoost, Least absolute shrinkage and selection operation (Lasso), Ridge, and Elastic network, were integrated into 43 algorithm combinations. Forty-three radiomics models were constructed separately using radiomics features extracted from arterial phase (AP), venous phase (VP), and combined arterial and venous phase (AP+VP) images. The concordance index (C-index) of each model was calculated. The model with the highest mean C-index was identified as the best model for calculating the radiomics score (Radscore). Univariate and multivariate Cox analyses were used to identify independent prognostic indicators and create a clinical model for prognosis prediction. The multivariable Cox regression was used to combine Radscore with clinical features to create a combined model. The efficacy of the model was evaluated using the C-index, calibration curves, and decision curve analysis (DCA).

**Results:**

The model based on the Lasso+StepCox[both] algorithm constructed using AP+VP radiomics features showed the best predictive ability among the 114 radiomics models. The C-indices of the model in the training and validation cohorts were 0.742 and 0.722, respectively. Based on the results of the univariate and multivariate Cox regression analyses, sex, Tumor-Node-Metastasis (TNM) stage, and systemic inflammation response index were included to build the clinical model. The combined model, incorporating three clinical factors and AP+VP-Radscore, achieved the highest C-indices of 0.764 and 0.746 in the training and validation cohorts, respectively. In terms of preoperative prognosis prediction for PDAC, the calibration curve and DCA showed that the combined model had a good consistency and greatest net benefit.

**Conclusion:**

A combined model of clinical features and AP+VP-Radscore screened using multiple ML algorithms has an excellent ability to predict the prognosis of PDAC and may provide a noninvasive and effective method for clinical decision-making.

## 1 Introduction

Pancreatic cancer is the seventh leading cause of malignant tumor-associated mortality worldwide and is generally associated with insidious onset and poor prognosis (1). It has the lowest 5-year overall survival (OS) rate (9%) among all malignancies (2). Pancreatic ductal adenocarcinoma (PDAC) is the most common kind of cancer that develops in the exocrine cells of the pancreas, accounting for approximately 95% of all pancreatic malignancies (3). Surgical resection at an early stage is a unique treatment strategy that allows a clinical cure. However, at the time of diagnosis, only 15–20% of patients are suitable for surgical treatment (4). Five-year OS of tumor removal is < 10%, but adjuvant chemotherapy after resection doubles the 5-year survival to approximately 16-21% (5). Identifying patients with PDAC at heightened risk is a significant challenge, allowing for disease prognosis anticipation and prompt adjustment of treatment plans.

The American Joint Committee on Cancer (AJCC) Tumor-Node-Metastasis (TNM) staging system is the primary prognostic evaluation tool for pancreatic cancer. Despite receiving the same treatment, the clinical outcomes of patients with PDAC at the same AJCC stage may differ (6). Therefore, there is an urgent need to identify reliable markers for direct prognostic stratification and individualized care. There is increasing evidence that factors other than the intrinsic histopathological features of the tumor, such as host-related factors, may impact patients’ clinical outcomes (7). Inflammation linked to cancer has been proposed as the seventh hallmark of cancer (8). Numerous epidemiological studies have suggested that inflammation may play a significant role in the initiation and progression of PDAC (9). Recent studies on the tumor microenvironment (TME) have highlighted the role of immune cell infiltration in PDAC development, metastasis, and evasion (10, 11). Increased levels of inflammatory indicators, such as the systemic inflammation response index (SIRI) and systemic immune-inflammatory index (SII), have been associated with poorer prognosis in PDAC (12, 13). Integrating TNM stage and inflammatory markers may have significant value in accurately predicting patient survival. Therefore, the role of inflammatory markers in predicting prognosis should not be ignored.

Radiomics is a new methodology that transforms medical images into extractable data by quantitatively extracting high-throughput characteristics (14). Computed tomography (CT) is currently the most commonly used imaging technique for pancreatic cancer (15). Based on contrast-enhanced CT (CECT), radiomics guidance for clinical decision-making can utilize patients’ clinical information more completely without increasing their financial burden. Machine learning (ML), a branch of artificial intelligence, is widely applied in disease prediction (16). The application of various ML algorithms for disease risk prediction has become a hotspot in medical big data research (17). The integration of ML techniques with radiomics has shown considerable efficacy in predicting cancer prognosis (18). This technology offers precise, dynamic, and nonintrusive approaches to personalized medicine. Multiple studies have indicated that radiomics can accurately predict the prognosis of various cancers including hepatocellular carcinoma (19), breast cancer (20), and colorectal cancer (21). There are no reports on the use of radiomics and various ML algorithms for prognosticating PDAC, although both ML and radiomics have been widely used for disease prognostication. Therefore, comparing different ML models based on radiomics could yield valuable insights.

The aim of this study was to conduct a comparative analysis of seven ML algorithms to build and validate CECT-based radiomics models for predicting PDAC prognosis. Furthermore, the radiomics score and clinical features were integrated to create a nomogram, strengthen the predictive capacity, and potentially contribute to follow-up plans and personalized therapy.

## 2 Materials and methods

### 2.1 Patients

**Figure 1:**
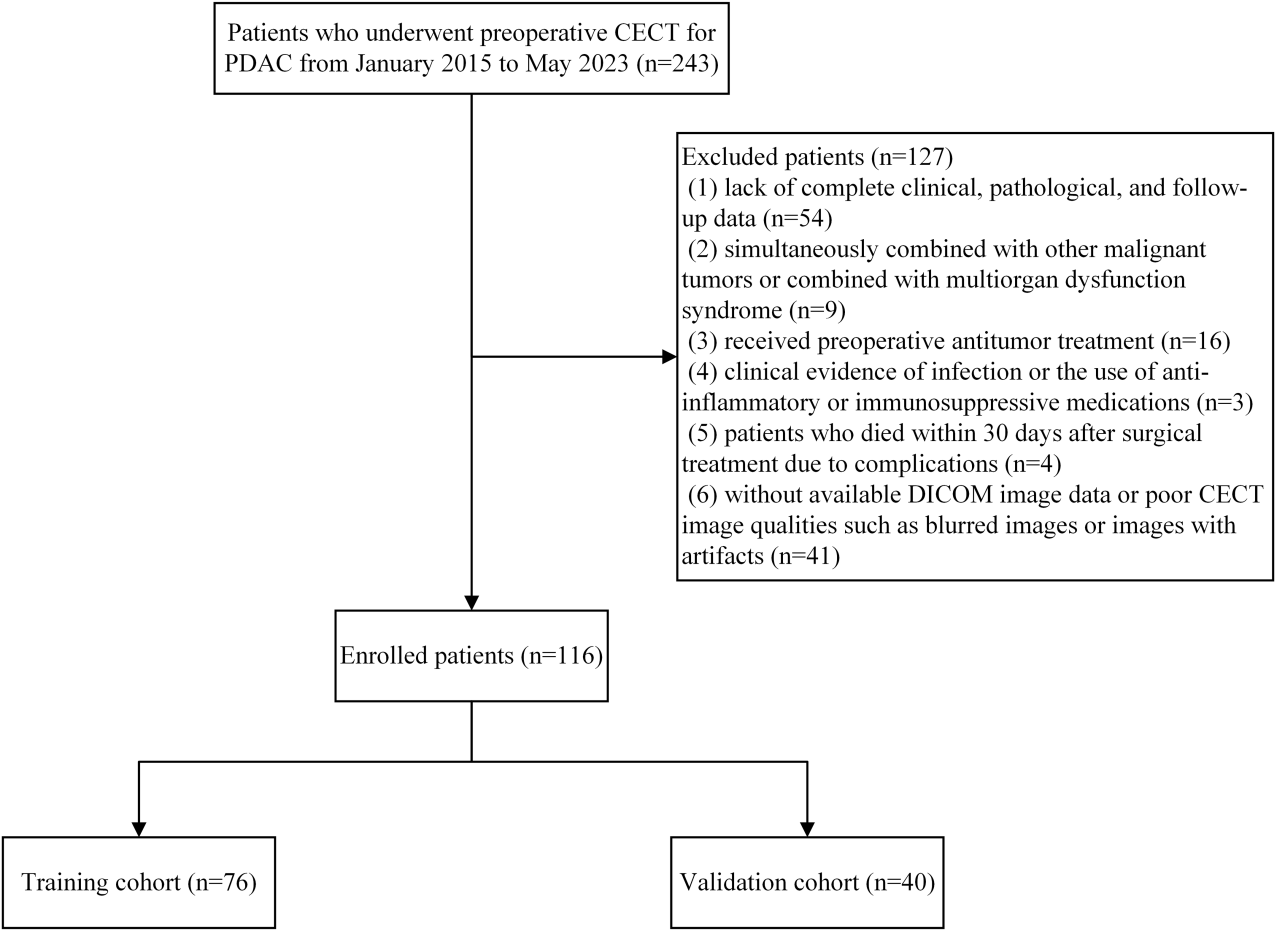
Flowchart depicting the patient selection process.

This retrospective study included patients with PDAC who underwent surgical resection at the Hepatobiliary and Pancreatic Surgery Department of the First Affiliated Hospital of Fujian Medical University between January 2015 and May 2023. The inclusion criteria were: (1) pancreatic ductal adenocarcinoma confirmed by postoperative pathology. (2) patients who underwent abdominal CECT within one month before surgery. Exclusion criteria were: (1) lack of complete clinical, pathological, and follow-up data; (2) simultaneously combined with other malignant tumors or combined with multiorgan dysfunction syndrome; (3) received preoperative antitumor treatment; (4) clinical evidence of infection or the use of anti-inflammatory or immunosuppressive medications; (5) patients who died within 30 days after surgical treatment due to complications; (6) without available digital imaging and communications in medicine (DICOM) image data or poor CECT image qualities such as blurred images or images with artifacts. For patients who underwent multiple preoperative CECT scans within one month before surgery, only the scan closest to the surgery date was used. This approach ensures that the imaging data most accurately represents the tumor’s status immediately prior to surgery. For patients with multiple preoperative laboratory test results, the data closest to the date of the selected CT scan were used. To ensure consistent application of the inclusion and exclusion criteria, two researchers independently reviewed the data for each patient. In cases of disagreement, a consensus was reached through discussion with a third researcher, ensuring uniform application of the patient selection criteria. A total of 116 PDAC cases were included and randomly separated into training and validation cohorts using the computer random number technique at a 2:1 ratio. This research was approved by the local Institutional Review Board and adhered to the Declaration of Helsinki guidelines. **Figure 1** shows the patient recruitment process and the inclusion and exclusion criteria.

### 2.2 Clinical data and follow up

Clinical data for this study were obtained from the electronic health record system, including age, sex, tumor location, TNM stage, carcinoembryonic antigen (CEA), carbohydrate antigen 19-9 (CA19-9), complete blood cell count, and biochemistry examination. Inflammatory and other indicators analyzed in this study were calculated using the blood tests mentioned above, including the SII, SIRI, albumin to fibrinogen ratio (AFR), aspartate aminotransferase/platelet ratio index (APRI), platelet to lymphocyte ratio (PLR), and prognostic nutritional index (PNI). All formulas are listed in **Supplementary Table 1**.

The primary outcome measure investigated in this study was OS, defined as the duration from the first diagnosis to either death or completion of the follow-up period. Follow-up was conducted by retrieving medical records from inpatients, outpatient visits, or telephone conversations. The follow-up period ranged from one to 97 months, with an average of 18.34 months, ending in September 2023.

### 2.3 CT image acquisition

Every patient underwent CT using either the Toshiba Aquilion One 320-slice spiral CT or the Toshiba Aquilion PRIME 80-slice spiral CT machine. Three-phase enhancement scans were performed in each patient. A high-pressure syringe was employed to inject the contrast material via the elbow vein at a 3.0 ml/s flow rate. Subsequently, 30 ml of normal saline was injected at 3.0 ml/s. Images were captured in the arterial, portal venous, and equilibrium phases at 30, 60, and 120 s, respectively, after injection of the contrast material. The following scanning settings were used: tube voltage, 120 kV; tube current, 230 mAs; rotation time, 0.35 seconds; and slice thickness, 5 mm.

### 2.4 Region of interest segmentation and feature extraction

For the analysis, the DICOM format was used after obtaining the preoperative CECT scans of all participants from the Picture Archiving and Communication Systems. One radiologist (reader 1) used the 3D Slicer (version 4.10.2) to manually delineate the region of interest (ROI) around the tumor margins on the arterial phase (AP) and venous phase (VP) CECT images. The images from 50 patients were randomly selected and the radiomics features of each ROI were extracted to calculate the intra- and inter-observer reliabilities. The ROI was segmented by reader 1 and a hepatobiliary surgeon (reader 2), who was blinded to the clinical information, to evaluate inter-observer reliability. The segmentations were repeated by reader 1 after one month to evaluate the intra-observer reliability. Before acquiring the radiomics characteristics, the scans were resampled to a voxel size of 1 × 1 × 1 mm^3^ to mitigate potential interference arising from variations in the equipment and scanning conditions. The “pyradiomics” package was used to extract radiomics characteristics from ROI to comply with the requirements outlined by the Image Biomarker Standardization Initiative (22).

### 2.5 Feature selection and radiomics model building

Z-score normalization was applied because of the varying means and variances across the different characteristics. The radiomics model was developed using reproducible, nonredundant, and informative candidate imaging features. First, the intraclass correlation coefficient (ICC) was used to assess the repeatability of the radiomics features within and between observers. The features selected for further investigation had ICCs >0.75. Next, univariate Cox regression analysis was performed to identify radiomics features that may have predictive value in the training cohort. To compare different ML algorithm effects and acquire a radiomics model with greater prediction ability, seven ML techniques were utilized, including supervised principal components (SuperPC), stepwise Cox, random survival forest (RSF), CoxBoost, least absolute shrinkage and selection operation (Lasso), ridge, and elastic network (Enet). One method selects the features, while another is used to construct the predictive model. The final prognostic model was deemed invalid if it included less than two features. Ultimately, 43 ML algorithm combinations were integrated (**Supplementary Table 2**). After computing the concordance index (C-index) for each model, the model with the highest mean C-index value was determined to be the most ideal model. For subsequent analysis, the best prognostic radiomics score (Radscore) was screened from the models constructed for the AP, VP, and combined arterial and venous phase (AP+VP).

### 2.6 Clinical model and combined model construction

Clinical and combined models were developed and compared with the radiomics models. Univariate and multivariate Cox analyses were used to identify independent prognostic indicators and create a clinical model for prognosis prediction. Using multivariable Cox regression, the Radscore was combined with independent prognostic markers to create a combined model.

### 2.7 Model evaluation

The C-index was used to assess the accuracy of the clinical, Radscore, and combined models. To assess the generalizability and stability performance of the model, 5-fold cross-validation was utilized. The dataset was randomly divided into five subsets. Four subsets were used for model training, while the remaining subset served as the validation set. This process was repeated five times to ensure that each subset was used as the validation set once. The predictive performance was evaluated by computing the mean and standard deviation of the C-index through 5-fold cross-validation. Using the combined model, a prognostic nomogram that enabled the quantitative assessment of individual patient survival was devised. Furthermore, a dynamic nomogram was constructed to simplify calculations and assist in clinical decision-making. The receiver operating characteristic (ROC) curves at the one- and two-year periods, which further assess the nomogram’s discriminating capacity, are produced using the survival forecast scores obtained from the nomogram. To assess the agreement between the predicted and observed survival probabilities of the combined model, a calibration curve was constructed by applying a bootstrap approach with 1000 repeats. Predicted probabilities were divided into approximately three equal groups and compared to observed outcomes. The 45-degree line represents perfect calibration, indicating perfect prediction accuracy when the calibration curve aligns with this line. The prognostic performance of the combined model was compared with that of the clinical and radiomics models using decision curve analysis (DCA). The clinical value of the combined model was examined by quantitatively calculating the net benefit at various threshold probabilities. The net benefit is calculated by subtracting the proportion of false positives (weighted by the threshold probability) from the proportion of true positives. The horizontal axis of the decision curve represents the risk threshold, while the vertical axis displays net benefit. The ‘all’ line assumes all patients receive treatment, while the ‘none’ line assumes no patients are treated. A greater area under the decision curve indicates higher clinical utility. The optimal cut-off value for the ROC curve was determined by maximizing the Youden index. Based on the optimal cut-off, individuals in the training and validation groups were classified into high- and low-risk categories using their nomogram scores. Kaplan-Meier survival curves were generated to compare survival outcomes between the two risk groups, and the log-rank test was used to assess statistically significant differences in survival between the high- and low-risk categories.

### 2.8 Statistical analysis

Statistical analyses were conducted using R software (version 4.3.1) and SPSS software (version 23.0). Continuous variables were analyzed using either the Wilcoxon rank test or the t-test. The Chi-square test or Fisher’s exact test was used to perform statistical comparisons of categorical variables. The “survival” package was used to perform Kaplan-Meier and multivariate Cox survival analyses. The calibration and ROC curves were plotted applying the “ rms” and “survivalROC” packages. The DCA was constructed applying the “ggDCA” package. Statistical significance was set at P < 0.05.

## 3 Results

### 3.1 Patient characteristics

Based on the inclusion and exclusion criteria, 116 patients with PDAC were included in this study, with 76 and 40 patients in the training and validation cohorts, respectively. The median follow-up period for all patients was 468.5 days (range: 35–2912 days). Specifically, the training cohort had a median follow-up of 481 days (range:82–2912 days), while the validation cohort had a median of 456 days (range: 35–2643 days). A total of 58 patients died during follow-up. There was no significant difference in the OS between the training and validation cohorts (P = 0.847, **Supplementary Figure 1**). The demographic information and clinicopathological characteristics are summarized in **Table 1**. There were no significant variations in the factors between the two cohorts.

**Table 1:** Clinical features of the two cohorts’ patients.

Variables	Training cohort (n=76)	Validation cohort (n=40)	p value
Age	63.61 ± 9.85	59.73 ± 10.56	0.052
BMI	21.52 ± 3.02	21.88 ± 2.82	0.528
Sex			0.094
Male	39(51.32)	27(67.50)	
Female	37(48.68)	13(32.50)	
Diabetes			0.200
Absent	46(60.53)	29(72.50)	
Present	30(39.47)	11(27.50)	
Tumor location			0.131
Head	49(64.47)	20(50.00)	
Body and tail	27(35.53)	20(50.00)	
TNM stage			0.798
Stage I	29(38.16)	12(30.00)	
Stage II	31(40.79)	20(50.00)	
Stage III	10(13.16)	5(12.50)	
Stage IV	6(7.89)	3(7.50)	
CEA	3.39(2.12-5.72)	3.40(2.18-5.86)	0.919
CA19-9	159.60(30.92-501.50)	178.00(36.51-653.45)	0.951
TBIL	14.95(8.90-124.60)	18.55(9.10-80.10)	0.765
AFR	12.21 ± 3.10	11.37 ± 3.49	0.188
APRI	0.36(0.20-0.91)	0.43(0.21-0.73)	0.807
PLR	146.91(117.10-200.55)	149.35(113.12-184.83)	0.697
PNI	47.91 ± 4.96	48.98 ± 5.41	0.287
SIRI	0.81(0.60-1.36)	0.65(0.48-1.33)	0.189
SII	548.94(398.21-791.45)	449.34(319.32-730.91)	0.082

BMI, body mass index; TBIL, total bilirubin.

### 3.2 Feature selection and radiomics model establishing

From the ROI, 1,316 radiomics characteristics were obtained in the AP image, 1,316 in the VP image, and 2,632 in the AP+VP image. The radiomics characteristics with ICC>0.75 in the AP, VP, and AP+VP were 655, 787, and 1,442, respectively. Univariate Cox analysis of AP, VP, and AP+VP radiomics features showed 181, 232, and 413 radiomics features, respectively, associated with prognosis. Next, the radiomics features identified by univariate Cox analysis that were associated with pancreatic cancer prognosis were incorporated into the integration algorithm. Forty-three combinations of algorithms were applied to construct predictive models in the training group. Notably, 42, 35, and 37 models were built using the radiomics characteristics of the AP, VP, and AP+VP images, respectively.

**Figure 2:**
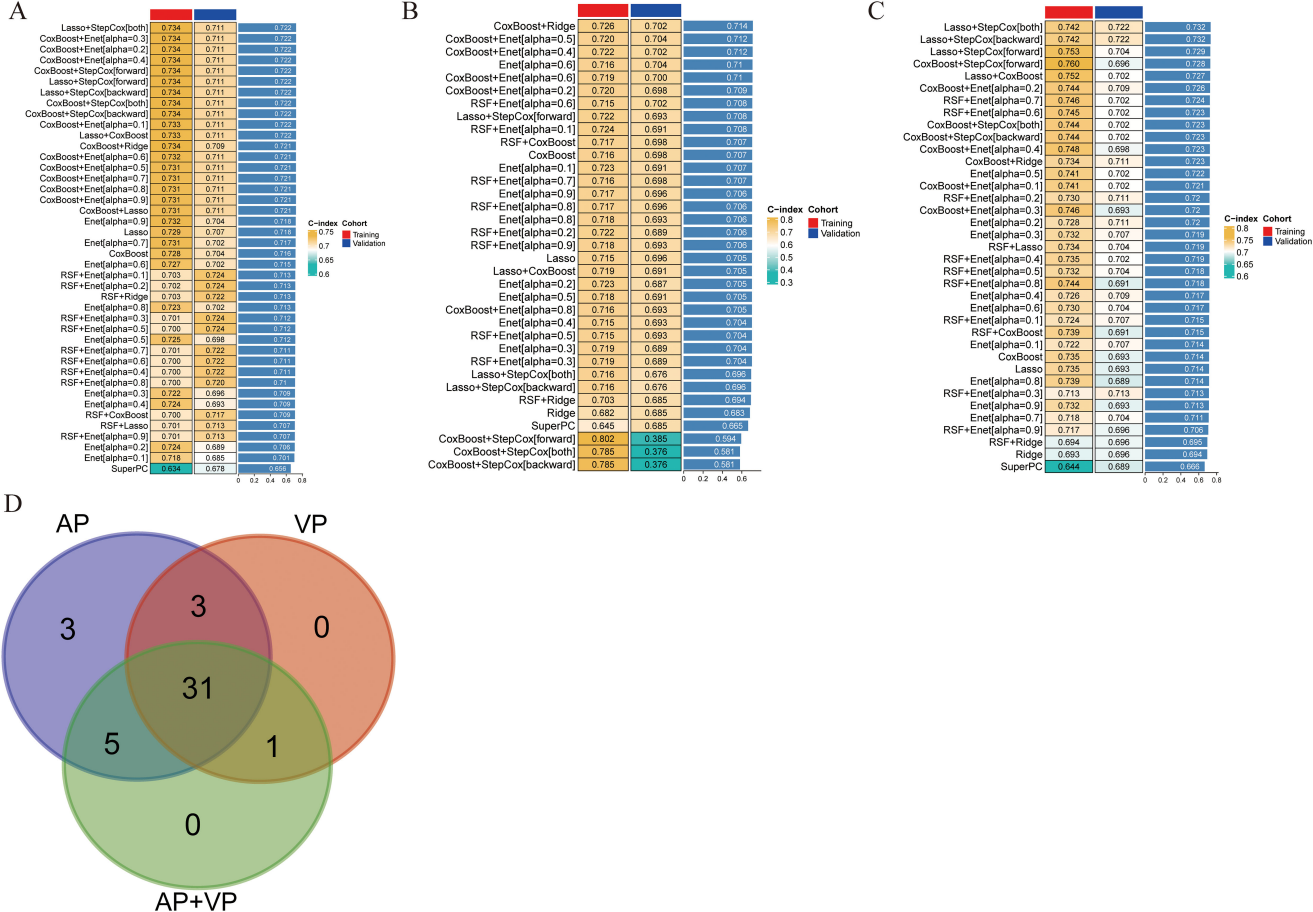
Predictive models were constructed in different phases of CECT, and the C-index was subsequently computed for each model. The transition from green to yellow signifies a progressive increase in the C-index value. Each column denotes a cohort, and each row denotes a model. **(A)** Arterial phase. **(B)** Venous phase. **(C)** Arterial phase + venous phase. **(D)** Wayne diagram of the number of models.

**Figure 3:**
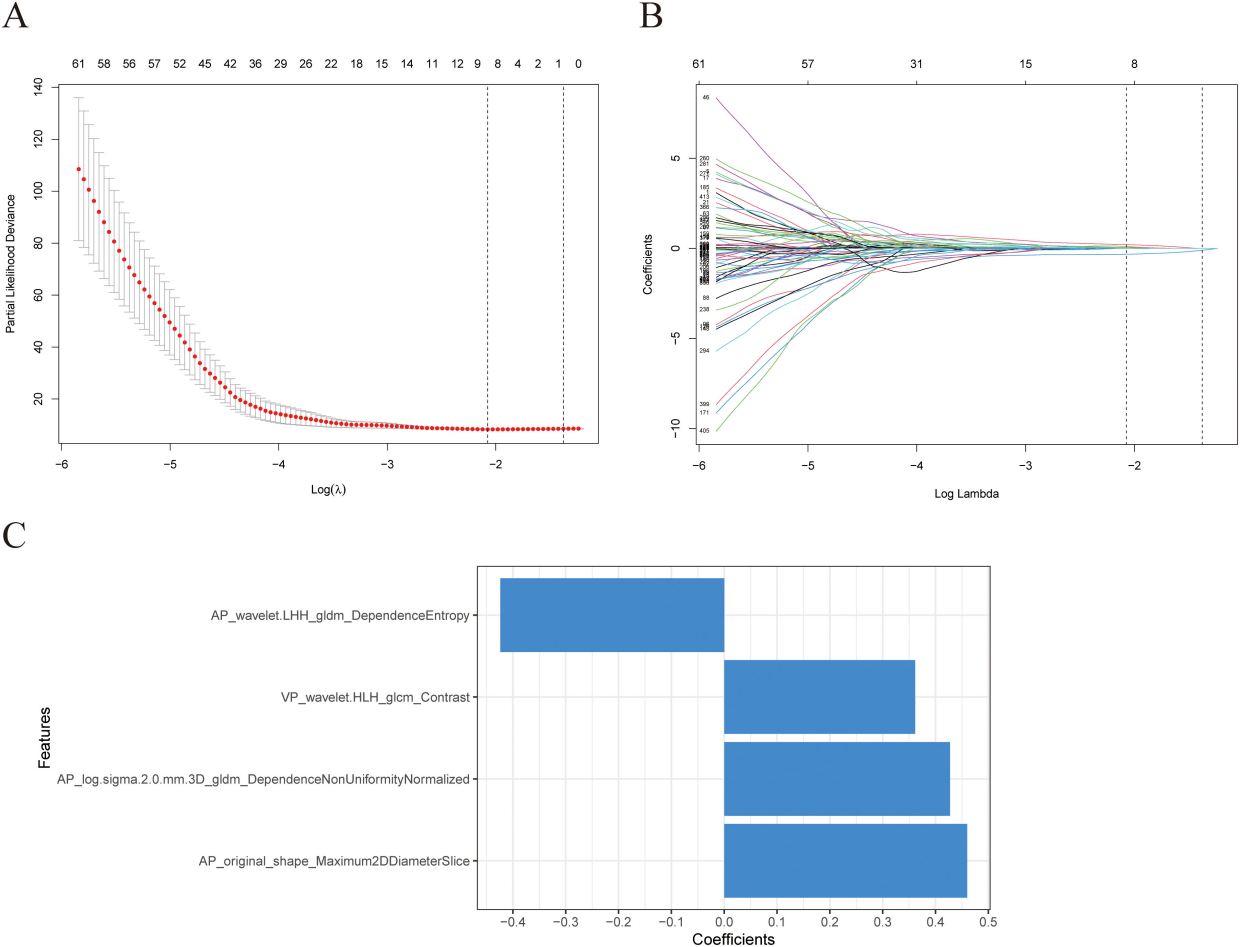
Selection of radiomics features using Lasso regression. **(A)** The model’s tuning parameters (lambda value) are chosen using the minimal criteria through ten-fold cross-validation. **(B)** Radiomics features’ Lasso coefficient profiles. **(C)** Information about the four features employed in this investigation and their respective feature weights.

The model constructed using the Lasso+StepCox[both] algorithm demonstrated a better prognostic prediction capacity in the AP models (**Figure 2A**). The Lasso+StepCox[both] model achieved C-indices of 0.734 and 0.711 in the training and validation cohorts, respectively. The model that exhibited the most favorable prognostic prediction performance among those developed using VP characteristics was produced using the CoxBoost+Ridge algorithm (**Figure 2B**). The C-indices of the CoxBoost+Ridge model in the training and validation cohorts were 0.726 and 0.702, respectively. Among the models developed using AP+VP characteristics, the Lasso+StepCox[both] algorithm produced the most accurate prognostic predictions (**Figure 2C**). The C-indices of the model were 0.742 and 0.722 in the training and validation sets, respectively. The model based on AP+VP radiomics characteristics, built using the Lasso+StepCox[both] algorithm, had the most effective prognostic prediction performance among the models developed with AP, VP, and AP+VP features. **Figure 2D** shows a Wayne diagram of the number of models constructed from the radiomics features of the AP, VP, and AP+VP images. Eight features with nonzero coefficients were chosen using Lasso from the training group (**Figures 3A, B**). Four radiomics features and their coefficients associated with pancreatic cancer prognosis were obtained using StepCox[both] regression analysis (**Figure 3C**).

### 3.3 Clinical model and combined model construction

Univariate Cox regression analysis was used to identify the clinical characteristics associated with OS in patients with PDAC (**Table 2**). Univariate analysis demonstrated that sex, TNM stage, and SIRI were significantly correlated with the overall survival of patients with PDAC in the training group. Subsequently, multivariate Cox regression analysis included significant variables identified in the univariate analysis. The findings show that sex (hazard ratio, HR=2.157(1.025-4.538), P=0.043), TNM stage (HR=1.821(1.269-2.613), P=0.001), and SIRI(HR=1.291(1.003-1.663), P=0.048) were identified in the multivariable analysis as independent indicators of survival for patients with PDAC (**Table 2**). A clinical model was created using independent prognostic factors, and clinical scores were determined for each patient. The AP+VP-Radscore was integrated with clinical variables, such as sex, TNM stage, and SIRI, to create a combined model.

**Table 2:** Conducting both univariate as well as multivariate Cox analyses on the clinical characteristics.

Characteristics	Univariate analysis		Multivariate analysis	
	Hazard ratio (95% CI)	P value	Hazard ratio (95% CI)	P value
Age	1.027(0.991-1.064)	0.140		
BMI	0.888(0.780-1.011)	0.072		
Sex	2.740(1.348-5.569)	0.005	2.157(1.025-4.538)	0.043
Diabetes	1.652(0.856-3.190)	0.135		
Tumor location	1.201(0.576-2.503)	0.625		
TNM stage	1.927(1.334-2.784)	<0.001	1.821(1.269-2.613)	0.001
CEA	0.997(0.983-1.012)	0.733		
CA19-9	1.001(1.000-1.002)	0.059		
TBIL	1.001(0.998-1.004)	0.469		
AFR	0.977(0.875-1.092)	0.683		
APRI	1.082(0.976-1.200)	0.133		
PLR	1.000(0.996-1.004)	0.976		
PNI	0.972(0.909-1.040)	0.415		
SIRI	1.320(1.043-1.670)	0.021	1.291(1.003-1.663)	0.048
SII	1.000(1.000-1.001)	0.437		

### 3.4 Model evaluation

**Figure 4:**
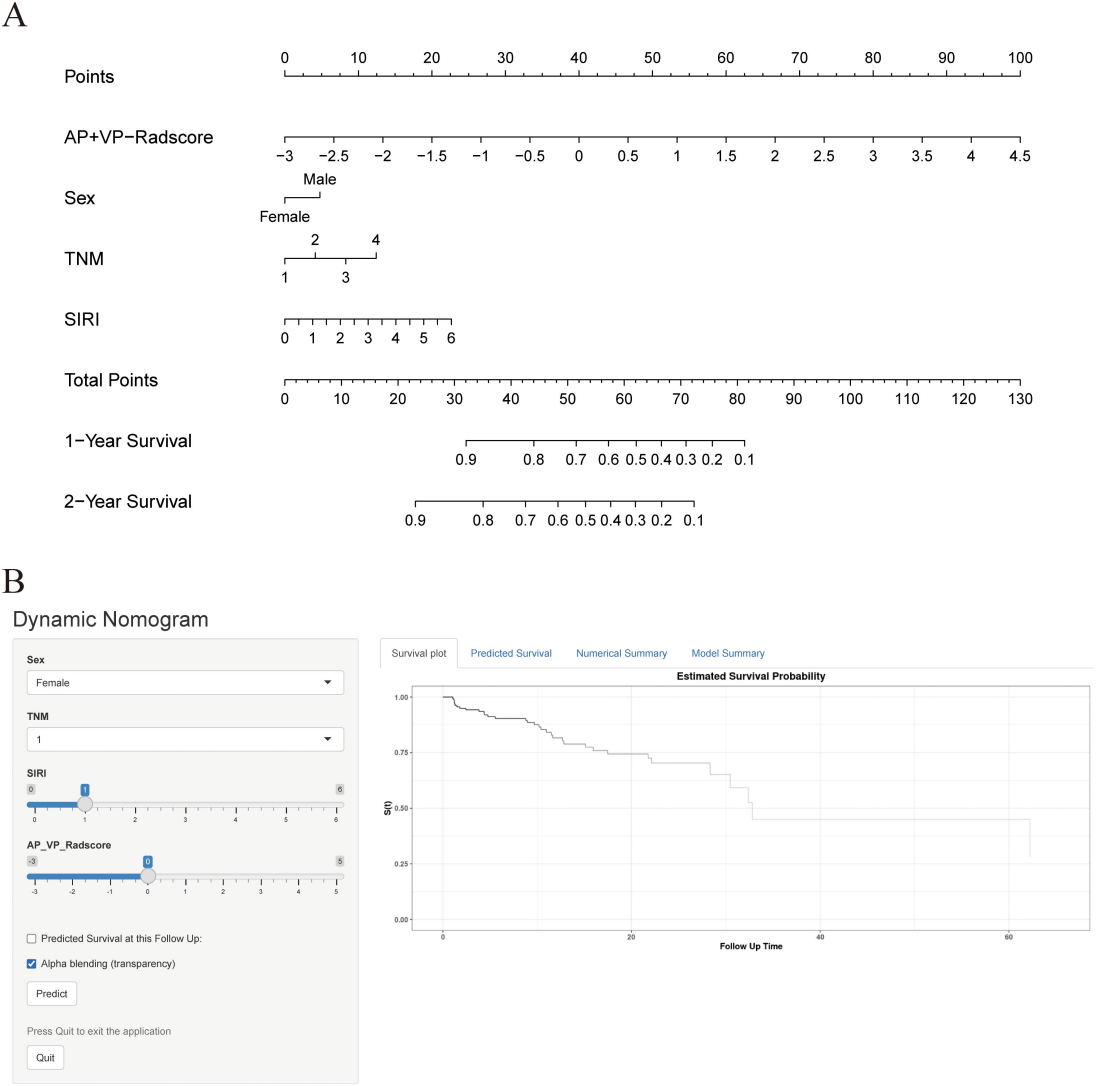
Construction of nomogram and dynamic nomogram. **(A)** Nomogram of the combined model for predicting the OS in patients with PDAC. **(B)** A dynamic nomogram for predicting the OS in patients with PDAC.

**Figure 5:**
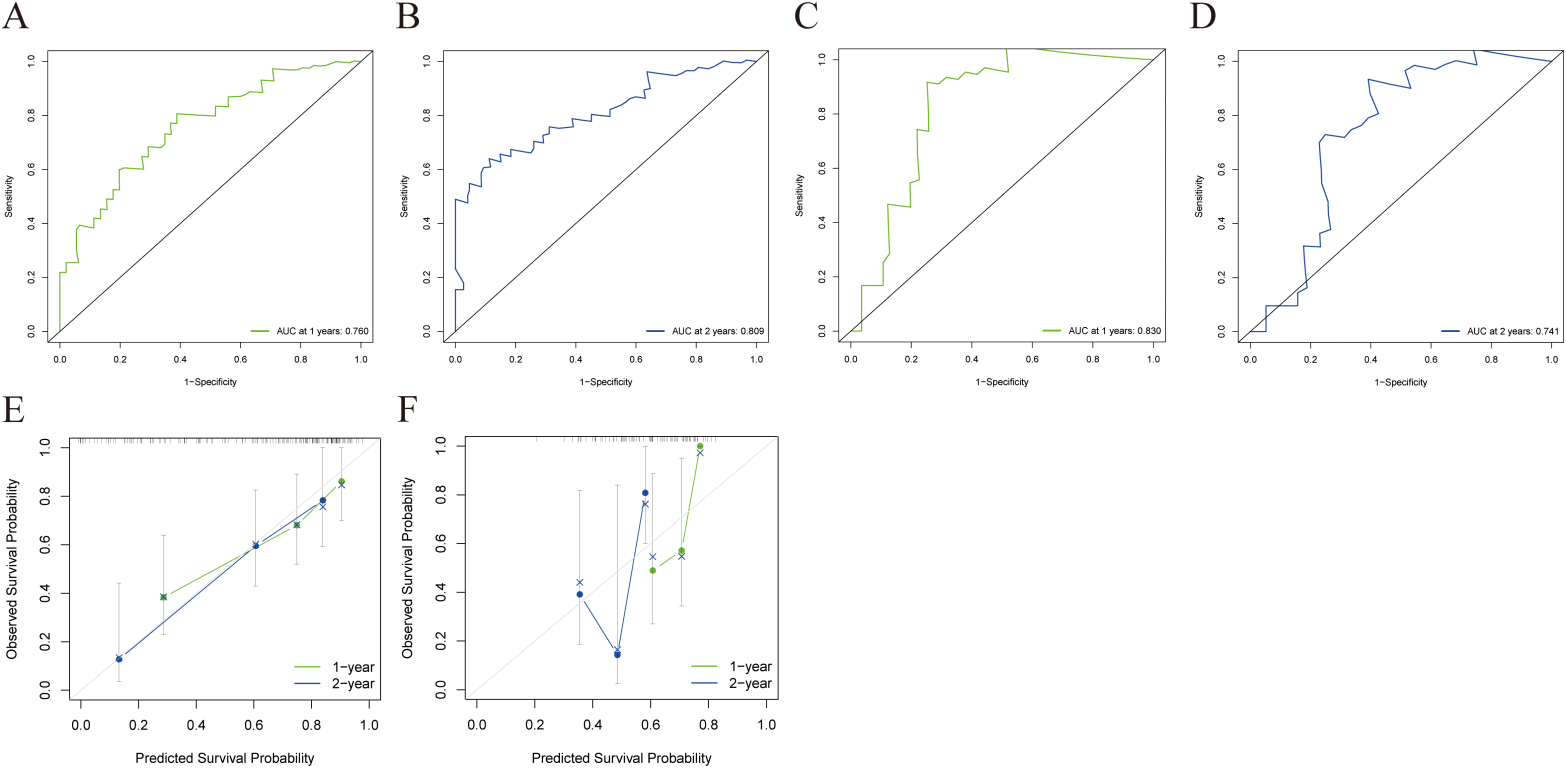
ROC and a calibration curve were used to assess the combined model’s predictive accuracy for 1- and 2-year OS in the training and validation cohorts. The combined model’s 1- **(A)** and 2-year **(B)** ROC curves for the training cohort. The combined model’s 1- **(C)** and 2-year **(D)** ROC curves for the validation cohort. The calibration curves of the combined model for the training **(E)** and validation cohorts **(F)**.

**Figure 6:**
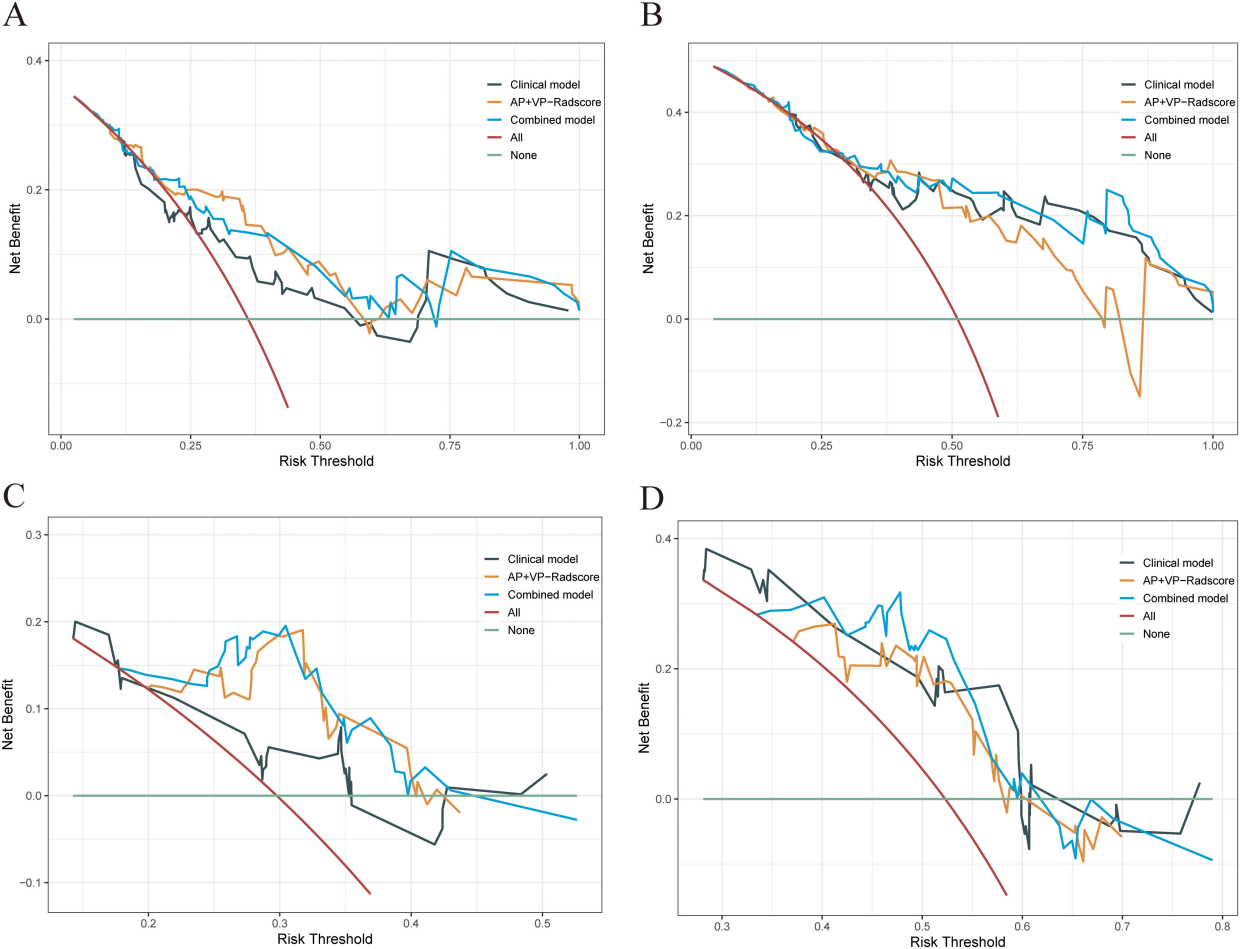
DCA was performed on each patient with PDAC to assess the clinical applicability of the combined model in predicting one- and two-year OS when compared to the clinical model and AP+VP-Radscore. The DCA curves of the clinical model, AP+VP-Radscore, and the combined model for predicting one- **(A)** and two-year **(B)** OS in the training cohort. The DCA curves of the clinical model, AP+VP-Radscore, and the combined model for predicting one- **(C)** and two-year **(D)** OS in the validation cohort.

**Figure 7:**
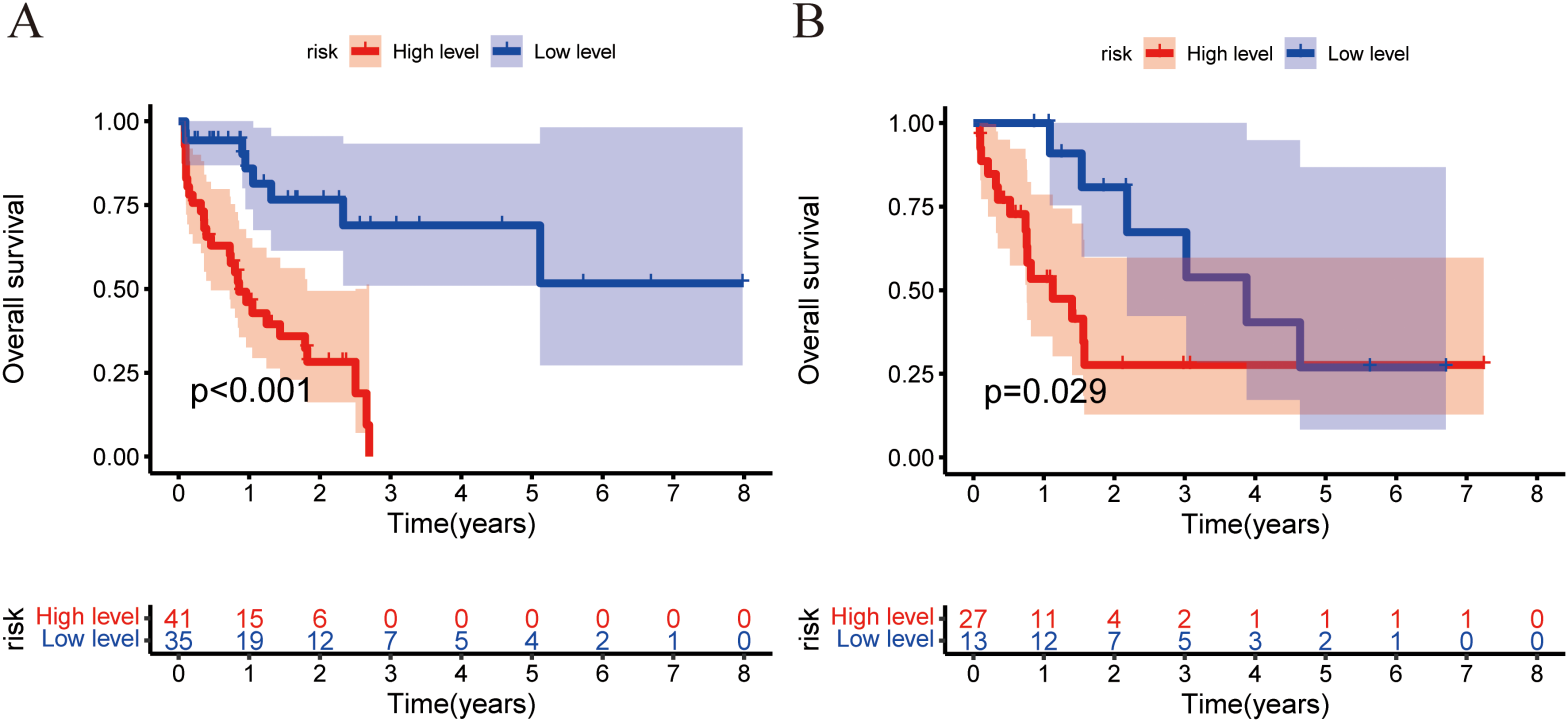
Training and validation cohorts used the Kaplan–Meier analysis and the log-rank test to measure the nomogram’s capacity for risk stratification. The Kaplan–Meier curves depicting the OS rates of the low-risk and high-risk groups were compared in the training **(A)** and validation **(B)** cohorts.

The combined model outperformed the clinical model and AP+VP-Radscore in predicting OS for PDAC, achieving the highest C-indices of 0.764 (95%CI: 0.683-0.846) and 0.746 (95%CI: 0.617-0.875) in the training and validation cohort, respectively (**Table 3**). The model’s generalization performance was evaluated using a 5-fold cross-validation approach. The combined model showed robust predictive performance in both the training (mean C-index ± standard deviation [SD], 0.751 ± 0.020) and validation (mean C-index ± SD, 0.752 ± 0.081) cohorts. Additionally, the C-index for each fold of the training and validation cohorts for the clinical, radiomics, and the combined model can be found in **Supplementary Table 3**. The combined model was visualized to form a nomogram (**Figure 4A**). To facilitate use by researchers and clinicians, an online dynamic nomogram (https://yuehuang.shinyapps.io/PDAC_OS_Nomogram/) was constructed based on the combined model (**Figure 4B**). **Figures 5A–D** displays the combined model for predicting one- and two-year OS (AUC = 0.760 and 0.809, respectively, in the training group; AUC = 0.830 and 0.741, respectively, in the validation group). Calibration curves of the combined model exhibited a high level of concordance between the predicted and observed survival probabilities (**Figures 5E, F**). According to the DCA curves, the combined model provided a higher net benefit than the clinical model or AP+VP-Radscore within a reasonable threshold probability range (**Figure 6**). Patients were divided into high and low nomogram score groups based on the cut-off value corresponding to the maximum Youden index. In the training cohort, patients with higher nomogram scores had a significantly poorer prognosis compared to those with lower nomogram scores, and this finding was validated in the validation cohort (**Figure 7**).

**Table 3:** Model performance in training and validation cohorts.

Model	Training cohort		Validation cohort	
	C-index	95% CI	C-index	95% CI
Clinical model	0.705	0.612-0.798	0.628	0.517-0.739
AP+VP-Radscore	0.742	0.653-0.831	0.722	0.591-0.853
Combined model	0.764	0.683-0.846	0.746	0.617-0.875

## 4 Discussion

In this study, multiple CECT-based radiomics models were established and validated to predict PDAC prognosis. Among the 114 radiomics models, the AP+VP radiomics model constructed using the Lasso + StepCox [both] algorithm performed best in predicting PDAC prognosis. By integrating clinical and radiomics features, the combined model outperformed the single clinical or radiomics models in terms of predictive performance. The combined model achieved acceptable results for both the training and validation cohorts. To the best of our knowledge, this is the first study to compare multiple ML algorithms combined with clinical features and radiomics features to predict PDAC prognosis.

As precision medicine advances, precise prediction of patient prognosis has been recognized as a fundamental element in tailoring treatment to individuals and enhancing patient outcomes. In a number of tumors, intratumor heterogeneity has been found to be prevalent and is associated with clinical outcomes (23). However, routine clinical examinations provide limited information concerning intratumor heterogeneity. Radiomics, as a noninvasive technique, can investigate potential phenotypic information concealed in images that is not apparent to the human eye, thus improving diagnosis and assisting in individualized treatment strategies. There have been many investigations into the use of radiomics to estimate survival rates in many cancer types such as esophageal neuroendocrine carcinoma, hepatocellular carcinoma, colorectal cancer, and clear cell renal cell carcinoma (19, 21, 24, 25). Compared with conventional radiological features, radiomics may provide thorough details about intratumor heterogeneity that radiologists may not be able to observe with the naked eye. Radiomics partially addresses constraints associated with conventional radiology. CECT is a commonly utilized method for evaluating PDAC (26). Previous studies have found that CT texture features are associated with survival outcomes in PDAC (27–31). These studies only considered textural features; however, other radiomics features were neglected, which may not maximize the benefits of radiomics. For instance, a previous study developed a model to predict OS using texture features combined with CA19-9, achieving a C-index of 0.69 (31). Although this study provided valuable insights, the model primarily focused on texture features, potentially overlooking other important radiomic characteristics. In contrast, our AP+VP radiomics model incorporates more comprehensive radiomic features, which may help capture additional predictive information, resulting in a higher C-index of 0.722. Most prior investigations on the prognosis of PDAC solely focused on segmenting the tumor image with the largest cross-sectional area to gather radiomics features, which failed to completely depict the tumor heterogeneity (29, 32). Compared to 2D segmentation, 3D segmentation provides more comprehensive information about the tumor. In previous studies, feature selection and modeling algorithms were primarily determined by scholars based on their preferences and knowledge gaps (31–33). To address this limitation, seven ML algorithms that can be used to build radiomics models were gathered. In total, 43 ML algorithm combinations were eventually integrated. In the study by Wang et al. (33), their Rad-score achieved a C-index of 0.675 (95% CI: 0.594-0.755) in the validation cohort​. Additionally, our AP+VP radiomics model demonstrated a higher C-index of 0.722 (95% CI: 0.591-0.853) in the validation cohort. This improvement may be attributed to our approach of building and comparing multiple ML algorithms, which enhanced the predictive accuracy of the model. Park et al.’s study (34) used thin-slice CT and a comprehensive ROI delineation that included the background pancreas, capturing more information about the tumor and surrounding tissues. Their model demonstrated excellent performance in prognosis prediction, with a C-index of 0.7414, indicating a high predictive ability. While our study did not utilize thin-slice CT or include the background pancreas in the ROI, it incorporated clinical variables such as inflammatory markers, alongside 43 ML algorithm combinations. The C-index reached 0.764 in the training cohort and 0.746 in the validation cohort. Although the difference in C-index compared to Park et al.’s model is modest, the use of multiple ML algorithms and inflammatory markers contributed to improving the predictive performance of the combined model in prognosis prediction. Unlike previous studies, we segmented the tumors layer-by-layer and extracted complete radiomics features to thoroughly investigate tumor heterogeneity. A total of 114 radiomics models were developed using dimensionality reduction techniques and classifiers. The efficacy of each model was compared, and the optimum algorithm combination was determined. This was the highlight of this study.

Among the AP, VP, and AP+VP radiomics models, the AP+VP-Radscore constructed by combining the LASSO and StepCox algorithms had the highest average C-index (0.732). LASSO uses L1 regularization to effectively select radiomic features related to prognosis from high-dimensional data, while also helping to prevent overfitting. This is particularly important in radiomics research, where the large number of features and potential multicollinearity can complicate analysis. The StepCox model, known for its robust performance in survival analysis, efficiently assesses the relationship between features and survival time. By integrating LASSO for feature selection and StepCox for model construction, we simplified the model while retaining key variables that contribute significantly to prognosis, thus improving the model’s stability and predictive performance. This algorithm combination not only provides strong interpretability but also has the potential to support clinical decision-making, highlighting the significance of radiomic features in cancer prognosis.

The AP+VP radiomics model predicted the OS with the highest C-indices among the AP, VP, and AP+VP radiomics models. A possible reason for this result is that the combined AP and VP radiomics features would contain more information about tumor heterogeneity than single-phase images. Several edge detection techniques are often used to conduct in-depth picture analysis and studies, including wavelet filters and the Laplacian of Gaussian (LoG) filters (35). The wavelet and LoG features have been shown to provide detailed information about tumor heterogeneity (36). Wavelet transformation has favorable properties for capturing local features and providing high-dimensional radiomics features that may not be visible to the human eyes. Wavelet functions provide strong antinoise properties at large scales and robust image detail extraction properties at smaller scales. Hence, it is possible to attain a harmonious equilibrium between noise suppression and extraction of picture edge features. In contrast to subjective assessments by radiologists and low-dimensional features, the utilization of wavelet features offers a greater wealth of information about the biological behavior and heterogeneity observed in different tumor types, such as intrahepatic cholangiocarcinoma (37), renal cell carcinoma (38), and prostate cancer (39). Previous research has shown that wavelet characteristics may measure intratumor heterogeneity and correlate with prognosis (40). Jiao et al. observed a correlation between wavelet characteristics and poor outcomes in individuals with early stage lung cancer (41). This highlights the significance of wavelet filters in radiomics investigations. The LoG comprises Laplacian and Gaussian kernels. The Laplacian kernel exhibits sensitivity towards areas characterized by rapid variations in intensities, accentuating specific textural details in the original image, and enhancing edges. Applying a Gaussian smoothing filter prior to the Laplacian operation is typically necessary to minimize the image’s sensitivity to noise. In the Cox analysis using the step method, we found that the features from the image transform type of the LoG-AP_log.sigma.2.0.mm.3D_gldm_DependenceNonUniformityNormalized was an independent prognostic variable for PDAC (P < 0.05). Findings from this study indicate that feature filtering by LoG enhances the identification of high-risk patients with poor prognosis in PDAC. The filtering method effectively captures heterogeneity in spatial pixel distributions, enhancing the biological relevance of radiomic features. We observed a significant correlation between the radiomic features AP_original_shape_Maximum2DDiameterSlice and PDAC prognosis. This feature demonstrates the size of the tumor region in AP images. Many studies have identified tumor size as an independent risk factor for PDAC prognosis. According to previous research, the early detection of small pancreatic adenocarcinomas (tumor size < 4.0 cm) is vital for improving prognosis (42). One study indicated that tumor size is an autonomous prognostic factor after surgical treatment (43). One potential explanation for this phenomenon is that larger tumors are more likely to invade adjacent tissues and metastasize to regional lymph nodes.

Local immune responses and systemic inflammation are important in the initiation and progression of several solid malignancies (7). Systemic inflammation involves cytokines, small inflammatory proteins, and immune cells, which circulate and are measurable in the bloodstream (44). Tumor-secreted proteins can influence the bone marrow microenvironment, leading to enhanced production of myeloid cells. During inflammation, it is possible to release neutrophil precursors, including myelocytes and promyelocytes, thereby increasing circulating granulocytes (45). Neutrophils can assist tumor cells in evading immune monitoring and adhering to metastatic organs (46). Furthermore, they contribute to cancer-related angiogenesis by secreting proangiogenic factors (47). Neutrophils release substantial quantities of nitric oxide and reactive oxygen species, which contribute to T-cell activation disorders (48). Circulating monocytes are a source of tumor-assisting macrophages (TAMs), which may be attracted to the tumor site and facilitate cancer cell proliferation and migration (49). Furthermore, they promote programmed cell death of activated T-cells and expedite tumor neovascularization (50). To a certain degree, the monocyte levels in the peripheral circulation may indicate the abundance of TAMs. Lymphocytes play an important role in the immune system, acting as a primary defense against cancer cells. They inhibit tumor growth by secreting cytokines, like interferons and tumor necrosis factor (51). A reduction in peripheral lymphocytes weakens the host’s anticancer immune response, leading to a higher risk of tumor cell spread. These cell types combine to form the SIRI, capturing the intricate interactions involving inflammatory and immune cells within the TME. Recent reports have shown that the SIRI can potentially be a prognostic indicator for survival outcomes across various tumor types (7, 52, 53). In this study, SIRI served as an independent prognostic marker for PDAC. SIRI has demonstrated the potential to improve prognostic accuracy in patients with PDAC and assist physicians in post-surgical monitoring and treatment planning.

Several studies have shown that sex is a non-negligible factor in the prognosis of patients with PDAC. Studies have shown that sex correlates with pancreatic cancer prognosis (54). Akira et al. identified being male as an independent predictive variable for PDAC prognosis (55). Similarly, findings from this study indicated that sex is an independent predictive factor for OS in PDAC. Nutritional status has been shown to correlate with disease prognosis and is essential in patients with cancer (56, 57). Preoperative malnutrition is significantly correlated with suboptimal postoperative wound healing and increased occurrence of complications (58). The PNI is used to evaluate nutritional and immunological conditions in individuals undergoing surgery. Previous studies have shown that the PNI is an independent factor for OS rates among individuals who have undergone surgical resection for PDAC (59). However, this investigation found no significant association between PNI and PDAC prognosis, likely due to differences in the populations included in this study. A prospective investigation with a large patient population is required to validate the predictive value of PNI in PDAC.

The current gold standard for predicting prognosis of PDAC is the TNM classification system developed by the AJCC (60). The findings of this study demonstrated that TNM stage was an independent predictive variable using multivariate Cox regression analysis. TNM stage classification focuses on the anatomic level of the lesion; however, individuals with the same stage may have varied prognoses (61). This drawback has the potential to result in either excessive or insufficient treatment. The integration of clinical features and imaging data is necessary to improve individualized prognostic prediction and application of precision therapy. The degree of tumor heterogeneity, which is closely reflected in imaging data, is a crucial sign of a tumor’s proliferative and metastatic potential. In this study, we mine the imaging data through radiomics. In terms of clinical data, we incorporated demographic factors, inflammatory markers, and nutritional indicators to comprehensively assess host-related characteristics and their potential influence on tumor progression and prognosis. Finally, we developed a combined model that integrated clinical features with CECT radiomics features. The validation findings demonstrated that compared with other models, the combined model performed better in terms of prognosis prediction. The forecasting efficacy of the combined model outperformed that of the clinical model (C-index: 0.746 versus 0.628). It may be related to the fact that more information about tumor heterogeneity and host-related factors was obtained by combining clinical and CECT image features. Subsequently, the improved net clinical benefit of the combined model was validated using DCA curves. These results suggest that models combining the AP+VP-Radscore and clinical features enhance the prediction accuracy of single radiomics or clinical models. A nomogram was developed to enhance the accuracy of individual patient prognostic predictions by offering a personalized prediction method. Improving individual outcomes prediction will facilitate patient counseling, tailoring treatment plans to their specific conditions, and effectively scheduling patient follow-up.

However, this study has some limitations. First, this investigation was conducted at a single center, without external validation, which may limit the generalizability of the findings. We will further validate these findings with external validation in future studies. Second, the sample size was relatively small, which reduced the credibility and generalizability of the model. A large-scale cohort is required to validate these findings. Third, this was a retrospective study, and patients with incomplete data were excluded, which may have introduced selection bias. Fourth, due to the limited number of patients who survived beyond 3 and 5 years in this study, we did not conduct an in-depth analysis of the 3-year and 5-year survival predictions. Larger sample sizes and longer follow-up periods will be needed in future studies to validate the model’s applicability for long-term survival predictions. Fifth, tumor segmentation was conducted using images with thick slices (5 mm), which may have excluded some finer characteristics. Sixth, only AP and VP images were included in this study; delayed-phase and unenhanced images were not analyzed. Finally, the correlation between radiomics characteristics and genomic or proteomics data still needs to be investigated. Hence, it is necessary to conduct additional prospective studies using a more diverse dataset to verify these findings.

In conclusion, the prognosis of PDAC can be predicted based on CECT radiomics features and clinical features combined with multiple ML algorithms. This may be an accurate and noninvasive approach for predicting PDAC prognosis, contributing to clinical decision-making.

## Data Availability

The original contributions presented in the study are included in the article/**Supplementary Material**. Further inquiries can be directed to the corresponding authors.
