# Supplementary material for: Comparison of multiple machine learning models for predicting prognosis of pancreatic ductal adenocarcinoma based on contrast-enhanced CT radiomics and clinical features
Source: Front Oncol. 2024 Nov 13;14:1419297. doi: 10.3389/fonc.2024.1419297 (PMC11598923; doi:10.3389/fonc.2024.1419297)
Supplement: Supplementary file 1 [file DataSheet1.docx]

Supplementary Material

**Supplementary Table 1.** Formulas for inflammatory markers and nutritional markers.

| Formula |
| --- |
| PNI= albumin + 5 × lymphocyte |
| SII= neutrophil × platelet / lymphocyte |
| AFR = albumin / fibrinogen |
| SIRI= neutrophil × monocyte / lymphocyte |
| PLR = platelet / lymphocyte |
| APRI = [(AST level / upper limit of normal of AST) ×100] / platelet |

**Supplementary Table 2.** Algorithm combinations.

| Algorithm combination |
| --- |
| CoxBoost |
| CoxBoost + Enet [alpha=0.1] |
| CoxBoost + Enet [alpha=0.2] |
| CoxBoost + Enet [alpha=0.3] |
| CoxBoost + Enet [alpha=0.4] |
| CoxBoost + Enet [alpha=0.5] |
| CoxBoost + Enet [alpha=0.6] |
| CoxBoost + Enet [alpha=0.7] |
| CoxBoost + Enet [alpha=0.8] |
| CoxBoost + Enet [alpha=0.9] |
| CoxBoost + Lasso |
| CoxBoost + Ridge |
| CoxBoost + StepCox [backward] |
| CoxBoost + StepCox [both] |
| CoxBoost + StepCox [forward] |
| Enet [alpha=0.1] |
| Enet [alpha=0.2] |
| Enet [alpha=0.3] |
| Enet [alpha=0.4] |
| Enet [alpha=0.5] |
| Enet [alpha=0.6] |
| Enet [alpha=0.7] |
| Enet [alpha=0.8] |
| Enet [alpha=0.9] |
| Lasso |
| Lasso + CoxBoost |
| Lasso + StepCox [backward] |
| Lasso + StepCox [both] |
| Lasso + StepCox [forward] |
| Ridge |
| RSF + CoxBoost |
| RSF + Enet [alpha=0.1] |
| RSF + Enet [alpha=0.2] |
| RSF + Enet [alpha=0.3] |
| RSF + Enet [alpha=0.4] |
| RSF + Enet [alpha=0.5] |
| RSF + Enet [alpha=0.6] |
| RSF + Enet [alpha=0.7] |
| RSF + Enet [alpha=0.8] |
| RSF + Enet [alpha=0.9] |
| RSF + Lasso |
| RSF + Ridge |
| SuperPC |

**Supplementary Table 3.** Predictive performance for the proposed models in five-fold cross validation.

| Folds | Model | Training cohort  C-index | Validation cohort  C-index |
| --- | --- | --- | --- |
| Fold 1 | Clinical model | 0.678 | 0.711 |
|  | AP+VP-Radscore | 0.758 | 0.683 |
|  | Combined model | 0.769 | 0.750 |
| Fold 2 | Clinical model | 0.680 | 0.806 |
|  | AP+VP-Radscore | 0.715 | 0.791 |
|  | Combined model | 0.730 | 0.842 |
| Fold 3 | Clinical model | 0.665 | 0.776 |
|  | AP+VP-Radscore | 0.726 | 0.755 |
|  | Combined model | 0.732 | 0.825 |
| Fold 4 | Clinical model | 0.698 | 0.617 |
|  | AP+VP-Radscore | 0.717 | 0.799 |
|  | Combined model | 0.751 | 0.671 |
| Fold 5 | Clinical model | 0.723 | 0.546 |
|  | AP+VP-Radscore | 0.739 | 0.678 |
|  | Combined model | 0.774 | 0.671 |

**
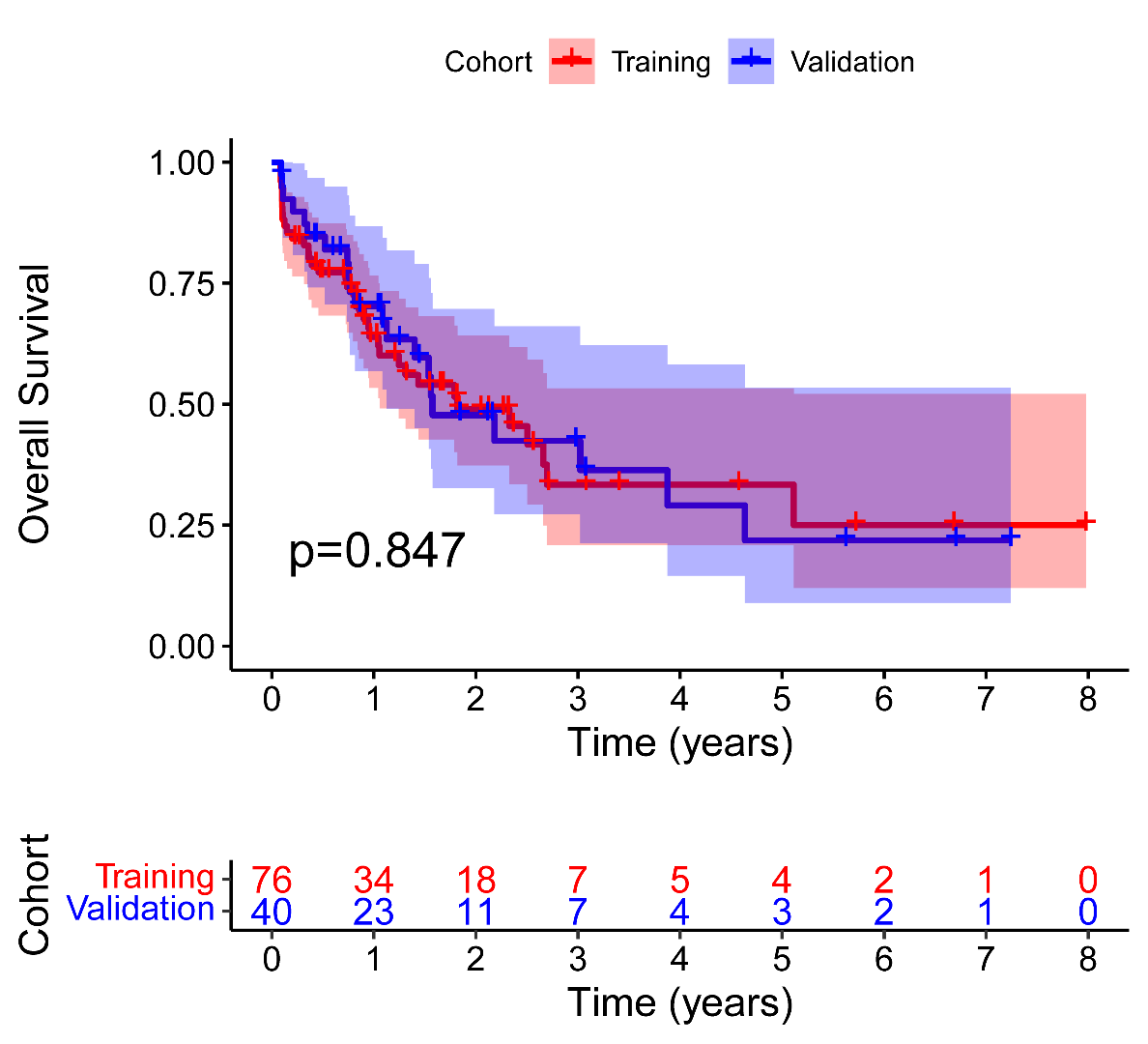
**

**Supplementary Figure 1.** Kaplan-Meier curves of OS in the training and validation cohorts.
